# Population-based projections of blood supply and demand, China, 2017–2036

**DOI:** 10.2471/BLT.19.233361

**Published:** 2019-10-18

**Authors:** Xiaochu Yu, Zixing Wang, Yubing Shen, Zhong Liu, Hongjie Wang, Shumei Zhang, Jia Gan, Fang Xue, Wei Han, Xin Shi, Yaoda Hu, Lei Wang, Ning Li, Peng Wu, Cuihong Yang, Jingmei Jiang

**Affiliations:** a Department of Nephrology, Peking Union Medical College Hospital, Chinese Academy of Medical Sciences, Beijing, China.; b Department of Epidemiology and Biostatistics, Institute of Basic Medical Sciences, Chinese Academy of Medical Sciences, No. 5, Dong Dan San Tiao, Dong Cheng District, Beijing 100005, China.; c Clinical Transfusion Research Center , Institute of Blood Transfusion, Chinese Academy of Medical Sciences, Chengdu, China.; d Beijing Red Cross Blood Center, Beijing, China.; e School of Statistics, Beijing Normal University, Beijing, China.; f Department of Blood Transfusion, Peking Union Medical College Hospital, Chinese Academy of Medical Sciences, Beijing, China.

## Abstract

**Objective:**

To estimate the long-term effect of the changing demography in China on blood supply and demand.

**Methods:**

We developed a predictive model to estimate blood supply and demand during 2017–2036 in mainland China and in 31 province-level regions. Model parameters were obtained from World Population Prospects, *China statistical yearbook 2016*, *China’s report on blood safety* and records from a large tertiary hospital. Our main assumptions were stable age-specific per capita blood supply and demand over time.

**Findings:**

We estimated that the change in demographic structure between 2016 (baseline year) and 2036 would result in a 16.0% decrease in blood supply (from 43.2 million units of 200 mL to 36.3 million units) and a 33.1% increase in demand (from 43.2 million units to 57.5 million units). In 2036, there would be an estimated shortage of 21.2 million units. An annual increase in supply between 0.9% and 1.8% is required to maintain a balance in blood supply and demand. This increase is not enough for every region as regional differences will increase, e.g. a blood demand/supply ratio ≥ 1.45 by 2036 is predicted in regions with large populations older than 65 years. Sensitivity analyses showed that increasing donations by 4.0% annually by people aged 18–34 years or decreasing the overall blood discard rate from 5.0% to 2.0% would not offset but help reduce the blood shortage.

**Conclusion:**

Multidimensional strategies and tailored, coordinated actions are needed to deal with growing pressures on blood services because of China’s ageing population.

## Résumé

**Objectif:**

Estimer l'effet à long terme de l'évolution démographique en Chine sur l'approvisionnement et la demande en produits sanguins.

**Méthodes:**

Nous avons élaboré un modèle de prévision pour estimer l'approvisionnement et la demande en produits sanguins en Chine continentale et dans 31 provinces entre 2017 et 2036. Les paramètres du modèle ont été définis à partir des Perspectives de la population mondiale, de l'*Annuaire statistique 2016 de la Chine*, du *Rapport de la Chine sur la sécurité transfusionnelle* et des dossiers d'un important hôpital de soins tertiaires. Nous sommes partis de l'hypothèse que l'approvisionnement et la demande en produits sanguins par habitant et par groupe d'âge étaient stables dans le temps.

**Résultats:**

Nous avons estimé que l'évolution de la structure démographique entre 2016 (année de référence) et 2036 entraînerait une diminution de l'approvisionnement en produits sanguins de 16,0% (de 43,2 millions d'unités de 200 mL à 36,3 millions d'unités) et une augmentation de la demande de 33,1% (de 43,2 millions d'unités à 57,5 millions d'unités). Nous estimons qu'en 2036, il manquera 21,2 millions d'unités. Une augmentation annuelle de l'approvisionnement de 0,9% à 1,8% est nécessaire pour maintenir un équilibre entre l'approvisionnement et la demande en produits sanguins. Cette augmentation n'est pas suffisante pour toutes les régions, car les différences régionales vont s'accentuer. Par exemple, un rapport demande/approvisionnement en produits sanguins ≥ 1,45 d'ici à 2036 est prévu dans les régions qui comptent un nombre élevé d'habitants âgés de plus de 65 ans. Les analyses de sensibilité ont montré qu'une augmentation des dons de 4,0% chaque année par des individus âgés de 18 à 34 ans ou une diminution du taux global de rejet de produits sanguins de 5,0% à 2,0% ne permettrait pas de réduire à néant la pénurie de produits sanguins, mais aiderait à la résorber.

**Conclusion:**

Des stratégies multidimensionnelles et des actions adaptées et concertées sont nécessaires pour faire face aux pressions croissantes qui pèsent sur les services de transfusion sanguine en raison du vieillissement de la population chinoise.****

## Resumen

**Objetivo:**

Estimar el efecto a largo plazo de los cambios demográficos en China sobre la oferta y la demanda de sangre.

**Métodos:**

Se desarrolló un modelo predictivo para estimar la oferta y la demanda de sangre durante el periodo en China continental y en 31 provincias. Los parámetros del modelo se obtuvieron de World Population Prospects (Perspectivas mundiales de población), *China statistical yearbook 2016 *(Anuario estadístico de China 2016), *China’s report on blood safety* (Informe de China sobre seguridad de la sangre) y de registros de un gran hospital terciario. Los supuestos principales eran la estabilidad de la oferta y la demanda de sangre per cápita específica de la edad a lo largo del tiempo.

**Resultados:**

Se estimó que el cambio en la estructura demográfica entre 2016 (año inicial) y 2036 resultaría en una disminución del 16,0 % en el suministro de sangre (de 43,2 millones de unidades de 200 ml a 36,3 millones de unidades) y un aumento del 33,1 % en la demanda (de 43,2 millones de unidades a 57,5 millones de unidades). En 2036, habría una escasez estimada de 21,2 millones de unidades. Se requiere un aumento anual de la oferta de entre el 0,9 % y el 1,8 % para mantener un equilibrio entre la oferta y la demanda de sangre. Este aumento no es suficiente para todas las regiones, ya que las diferencias regionales aumentarán, por ejemplo, se pronostica una relación entre la demanda y la oferta de sangre ≥ 1,45 para el año 2036 en regiones con grandes poblaciones mayores de 65 años. Los análisis de sensibilidad mostraron que el aumento de las donaciones en un 4,0 % anual por parte de las personas de 18 a 34 años o la disminución de la tasa general de descarte de sangre del 5,0 % al 2,0 % no compensaría sino que ayudaría a reducir la escasez de sangre.

**Conclusión:**

Se necesitan estrategias multidimensionales y acciones coordinadas y adaptadas para hacer frente a la creciente presión sobre los servicios de transfusión de sangre debido al envejecimiento de la población de China.

## ملخص

**الغرض:**

تقدير التأثير طويل الأجل للتغير الديموغرافي في الصين على العرض والطلب على الدم.

**الطريقة:**

لقد قمنا بتطوير نموذج تنبؤي لتقدير العرض والطلب على الدم خلال الفترة من 2017 إلى 2036 في البر الرئيسي للصين وفي 31 إقليماً. تم الحصول على المعاملات النموذجية من "التوقعات السكانية العالمية"، *الكتاب السنوي الإحصائي للصين لعام 2016 *، *تقرير الصين حول سلامة الدم *، وسجلات من مستشفى كبير فوق الثانوي. وكانت الافتراضات الرئيسية لدينا هي أن العرض والطلب على الدم يتميز بالاستقرار المرتبط بالعمر ومحدد حسب الفرد مع مرور الوقت.

**النتائج:**

لقد قدرنا أن التغيير في التكوين الديموغرافي بين عامي 2016 (سنة الأساس)، و2036 سيؤدي إلى انخفاض بمعدل 16.0% في عرض الدم (من 43.2 مليون وحدة تمثل 200 مل، إلى 36.3 مليون وحدة) وزيادة في الطلب بمعدل 33.1% (من 43.2 مليون وحدة إلى 57.5 مليون وحدة). وفي عام 2036، سيكون هناك نقصاً تقديرياً بقيمة 21.2 مليون وحدة. هناك حاجة لزيادة سنوية في العرض ما بين 0.9% و1.8%، وذلك للحفاظ على التوازن بين العرض والطلب على الدم. هذه الزيادة ليست كافية لكل منطقة حيث أن الاختلافات الإقليمية سوف تزدد، على سبيل المثال من المتوقع أن تبلغ نسبة العرض/الطلب في الدم 1.45 أو أكثر بحلول عام 2036 في المناطق التي بها نسبة سكانية كبيرة تبلغ أعمارها أكثر من 65 سنة. أظهرت تحليلات الحساسية أن زيادة التبرعات بنسبة 4.0% سنوياً بواسطة الأشخاص الذين تتراوح أعمارهم ما بين 18 و34 عاماً، أو انخفاض المعدل الكلي لإهدار الدم من 5.0% إلى 2.0% لن يحدث توازناً، ولكن يساعد في الحد من نقص الدم.

**الاستنتاج:**

هناك حاجة إلى استراتيجيات متعددة الأبعاد وإجراءات منسقة مصممة للتعامل مع الضغوط المتزايدة على خدمات الدم بسبب النسبة السكانية المسنة في الصين. ****

## 摘要

**目的:**

旨在估计中国人口结构变化对血液供需的长期影响。

**方法:**

我们设计了一个预测模型来估计 2017-2036 年中国大陆和 31 个省份的血液供需情况。模型参数取自《世界人口展望》、*《中国统计年鉴 2016》*、*《中国血液安全报告》*和某大型三级医院的记录。此次研究的基本假设是，随着时间的推移，特定年龄段的人均血液供需保持稳定。

**结果:**

我们估计，2016 年（基准年）至 2036 年间人口结构的变化将导致血液供应减少 16.0%【从 4320 万单位（每一单位血液为 200 毫升）降至 3630 万单位】，需求增加 33.1%（从 4320 万单位增至 5750 万单位）。到 2036 年，估计将产生 2120 万单位的血液短缺。需要每年增加 0.9% 到 1.8% 的供应量方可维持血液供需平衡。因为逐渐变大的地区差异，上述的增长速度还不足以满足各个地区的需求。例如，在大量人口超过 65 岁的地区，2036 年的预计血液供需比将达到≥1.45。敏感性分析表明，18-34 岁人群每年增加 4.0% 的献血量，或将总体血液报废率从 5.0% 降低到 2.0%，不会抵消但有助于缓解血液短缺的情况。

**结论:**

由于中国人口老龄化，需要多层面的战略和有针对性的协调行动来应对日益增长的血液供需压力。

## Резюме

**Цель:**

Оценить долговременный эффект демографических изменений в Китае на спрос и предложение на кровь.

**Методы:**

Авторы разработали модель прогнозирования для оценки спроса и предложения на кровь для материковой части Китая (31 провинция) в период 2017–2036 гг. Параметры модели были получены на основании обзора «Мировые демографические перспективы», *Статистического ежегодника для Китая за 2016 год*, *отчета Китая по безопасности крови* и документации крупной высокоспециализированной больницы. В качестве главного допущения авторы приняли устойчивый характер спроса и предложения на кровь в каждой определенной возрастной группе.

**Результаты:**

По оценкам авторов, изменения в демографической структуре в период с 2016 года (базовый уровень) до 2036 года приведут к сокращению предложения крови на 16% (с 43,2 млн порций по 200 мл до 36,3 млн порций) и к росту спроса на 33,1% (с 43,2 до 57,5 млн порций). Ожидается, что к 2036 году дефицит крови в стране достигнет цифры в 21,2 млн порций. Для поддержания баланса между спросом и предложением на кровь требуется ежегодное увеличение предложения крови на 0,9–1,8%. Такое увеличение предложения может оказаться недостаточным в определенных регионах, так как разница между регионами продолжит расти. Например, для регионов со значительной популяцией людей старше 65 лет к 2036 году прогнозируется коэффициент соотношения спроса и предложения ≥1,45. Анализ чувствительности показал, что увеличение донорства на 4% в год среди людей в возрасте от 18 до 34 лет или снижение общего показателя выбраковки донорской крови с 5,0 до 2,0% не сведет на нет нехватку крови, но поможет ее уменьшить.

**Вывод:**

Необходима разработка комплексных стратегий и принятие специализированных координированных мер для преодоления растущего давления на службы крови, связанного со старением населения Китая.****

## Introduction

Maintaining a safe and adequate supply of blood for health services is an important responsibility of every World Health Organization Member State.^1^^,^^2^ Demographic changes are a threat to the balance of blood demand and supply in blood services because both blood consumption and donation patterns vary by age, as has been shown in countries such as the United Kingdom of Great Britain and Northern Ireland,^3^ Germany,^4^ Canada^5^ and Japan.^6^ The challenge is even greater in China,^7^^,^^8^ which is the most populous country and is shifting towards an aged society.^9^ The proportion of the Chinese population aged 65 years or older is projected to double from 10% (142 million people) in 2016 to 22% (309 million) in 2036.^10^ The driving forces for this change are the combination of increased births during the 1960s and the sharp decrease in births in later generations because of the one-child policy that began in the 1970s and lasted more than 40 years.^11^ Although a universal two-child policy was started in 2016,^11^ China is projected to reach its peak population in 2029.^11^ As a result of these combined factors, shortages in blood supply are expected to increase for the next 20 years.

Blood shortages are already common in China because of the low blood donation rate,^12^ 10.5 donors per 1000 population in 2016. Despite a growth in the total volume of blood donation over 20 consecutive years in China, this rate is well below the world average of 30–40 donors per 1000 population.^13^ Regional variations in China’s population further add to difficulties in developing and coordinating blood services nationwide.^7^^,^^13^ The relatively affluent eastern part of China has long faced an ageing population.^14^ In underdeveloped and sparsely populated western China, which has 71% of China’s land area but only 28% of its population,^15^ life expectancy is below the national average of 76 years.^16^ A national survey in 2012–2014 also reported large regional variations in the ability of blood banks to supply blood.^17^

The objectives of our study were to: (i) estimate the differences between supply and demand of blood as a result of the changing demography in China by year and region and (ii) propose strategies to reduce these differences to ensure an adequate supply of blood for the needs of the Chinese population. 

## Methods

### Data sources

We estimated the annual supply of – and demand for – blood in mainland China between 2017 and 2036. We obtained the population sizes of different age groups in these years from World Population Prospects.^10^ We used the *China statistical yearbook* for 2016^18^ to extract age structure data of 31 provincial-level regions (22 provinces, five autonomous regions and four province-level municipalities) in the baseline year 2016.

We obtained data from *China’s report on blood safety 2016*^13^ on the overall supply and use of blood, age composition of blood donors, clinical use among specialties (e.g. surgery, obstetrics and gynaecology and intensive care), and rate of discarding blood (e.g. for physical reasons such as blood bag breakage, incomplete collection and haemolysed blood, disqualification because of positive test results for infections, for example human immunodeficiency virus and hepatitis B and C, and expiry). This report includes detailed data of blood services for mainland China, as well as the 31 province-level regions. We supplemented patient data on age, clinical specialty and blood use included in our previous study during 2015–2016^19^^,^^20^ with data from Peking Union Medical College Hospital, to estimate the age- and specialty-specific usage rate of blood. This hospital is a large tertiary facility and leading centre in blood transfusion therapy in China.

### Predictive model

The age criterion for donating blood in China is 18–59 years,^21^ and we divided this interval into five age groups (*n*) for the analysis: 18–24, 25–34, 35–44, 45–54 and 55–59 years. Similarly, we divided the population of potential blood users into nine age groups of 10-year spans (*m*): 0–9, 10–19, 20–29, 30–39, 40–49, 50–59, 60–69, 70–79, and ≥ 80 years. We used a calculation method (Box 1) to predict blood supply and demand by examining age-specific data on blood services and population projections, using the following formula.

Box 1 **Calculations for estimating blood supply and demand in China for 2017 to 2036**

**Prediction of supply**

1. Total blood supply in China in 2016 × Proportions of blood supply in different age groups in 2016 = Blood supply in different age groups in 2016
2. Blood supply in different age groups in 2016 ÷ Population in different age groups in 2016 = Per capita blood supply in different age groups
3. Per capita blood supply in different age groups × Population in different age groups in the forecast year = Blood supply in different age groups in the forecast year
4. The sum of blood supply in different age groups in the forecast year equals the total blood supply in the forecast year.

**Prediction of demand**

1. Total blood demand in China in 2016 × Proportions of blood demand in different age groups in 2016 = Blood demand in different age groups in 2016
2. Blood demand in different age groups in 2016 ÷ Population in different age groups in 2016 = Per capita blood demand in different age groups
3. Per capita blood demand in different age groups × Population in different age groups in the forecast year = Blood demand in different age groups in the forecast year
4. The sum of blood demand in different age groups in the forecast year equals the total blood demand in the forecast year.

Total blood supply in year *t* is
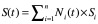
(1)where *N_i_*(*t*) is the population size of the *i*th age group in year *t* and *S_i_* is the per capita blood supply of the *i*th age group in the baseline year (2016), calculated as: *S_i_* = (*A* × *R_i_*)/*N_i_*(2016), where *A* is the overall blood supply in 2016 and *R_i_* is the proportion of blood supply of the *i*th age group in 2016.^13^

Similarly, total blood demand in year *t* is
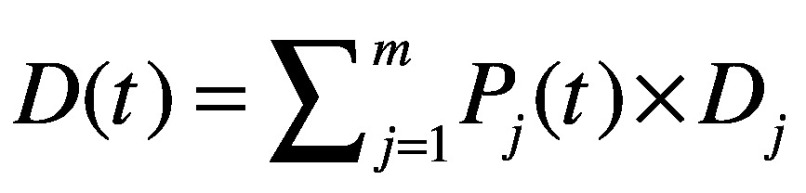
(2)where *P_j_*(*t*) is the population size of the *j*th age group in year *t* and *D_j_* is the per capita blood demand of the *j*th age group in 2016 calculated as: *D_j_* = (*A* × *Q_j_*)/*P_j_*(2016) where *A* is the overall blood supply in 2016 and *Q_j_* is the proportion of blood demand of the *j*th age group in 2016. These proportions were estimated by weighting the age structure of blood use in the Peking Union Medical College Hospital with the nationwide proportions of blood used by different specialties.^13^ No hospital can serve as a representative sample of blood use, therefore we validated this estimate using data from another report.^22^

### Assumptions

In a base-case scenario, we assumed that the supply and demand of blood per capita in each age group remains stable during the forecasted 20 years (2017–2036), as in the baseline year 2016.

As the actual need for blood cannot be determined,^23^^,^^24^ we assumed that blood demand in the baseline year was equal to supply (43.2 million units of 200 mL, including all blood components),^13^ and that this demand reflects appropriate use. 

By also assuming that the age-specific population growth ratios in each region were identical to national levels, we predicted the blood supply and demand for each of the 31 province-level regions, noting that China has a long-term shortage of blood.^12^

### Sensitivity analysis

We carried out the following sensitivity analyses to explore the effect of changing certain variables on blood supply. First, we lowered the current (2016) discard rate of blood of 5.0% to 2.0% throughout the country. The 2.0% rate was considered a reasonable forecast based on present achievable levels.^12^ Second, we increased the combined annual per capita blood donation from young people aged 18–24 and 25–34 years by 2.0% and by 4.0%. We selected these age groups because they are more physically fit for donation than older people, although few currently donate blood. Finally, we increased the overall annual blood supply for all age groups by 1.0% and 1.5%. We selected 1.0% and 1.5% based on actual annual growth rates of overall blood supply during 2012–2016.^13^

We used MATLAB R2018a (MathWorks^®^ Inc., Beijing, China) to analyse the data.

## Results

### Baseline blood supply and demand

**Fig. 1:**
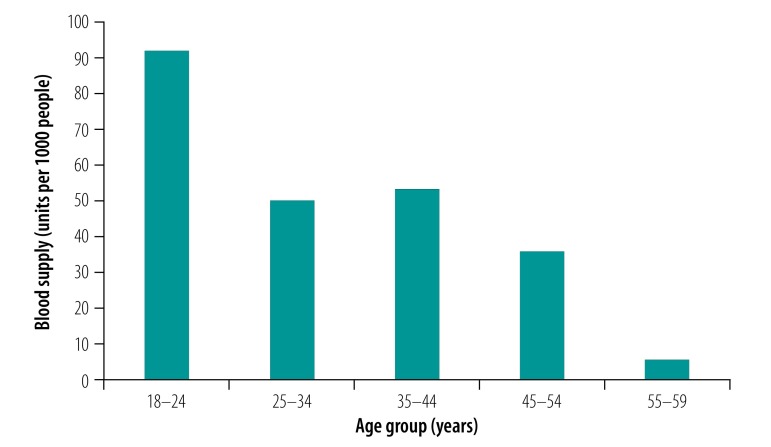
Blood donations by different age group in China in 2016, the baseline year

Note: One unit equals 200 mL blood.

**Fig. 2:**
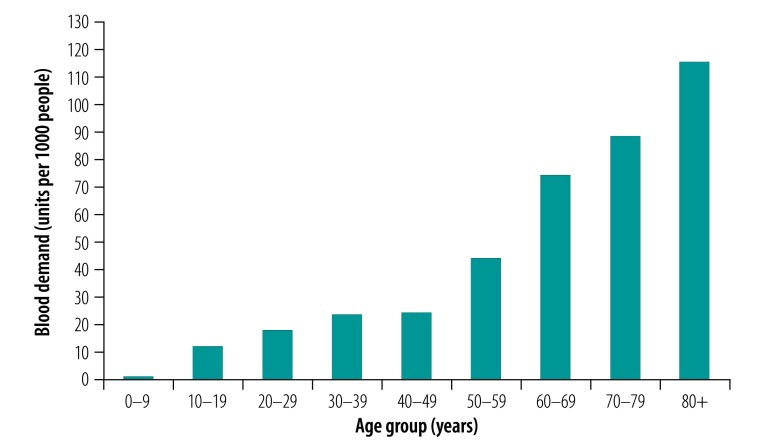
Blood consumption by different age group in China in 2016, the baseline year

Note: One unit equals 200 mL blood.

Fig. 1 shows the average amounts of blood (units per 1000 people) donated in 2016 for the different age groups. The youngest donation group (age 18–24 years) donated the most blood per 1000 people (91.8 units), followed by the age group 35–44 years (53.2 units) and the age group 25–34 years (49.9 units). The age group 55 years or older donated the smallest amount of blood (5.4 units). In contrast, the consumption of blood increased by age group (Fig. 2), from 1.0 unit per 1000 people in those aged 0–9 years to 43.9 units per 1000 people in those aged 50–59 years, and to as high as 115.3 units per 1000 people in those older than 79 years.

### Base-case predictions

**Fig. 3:**
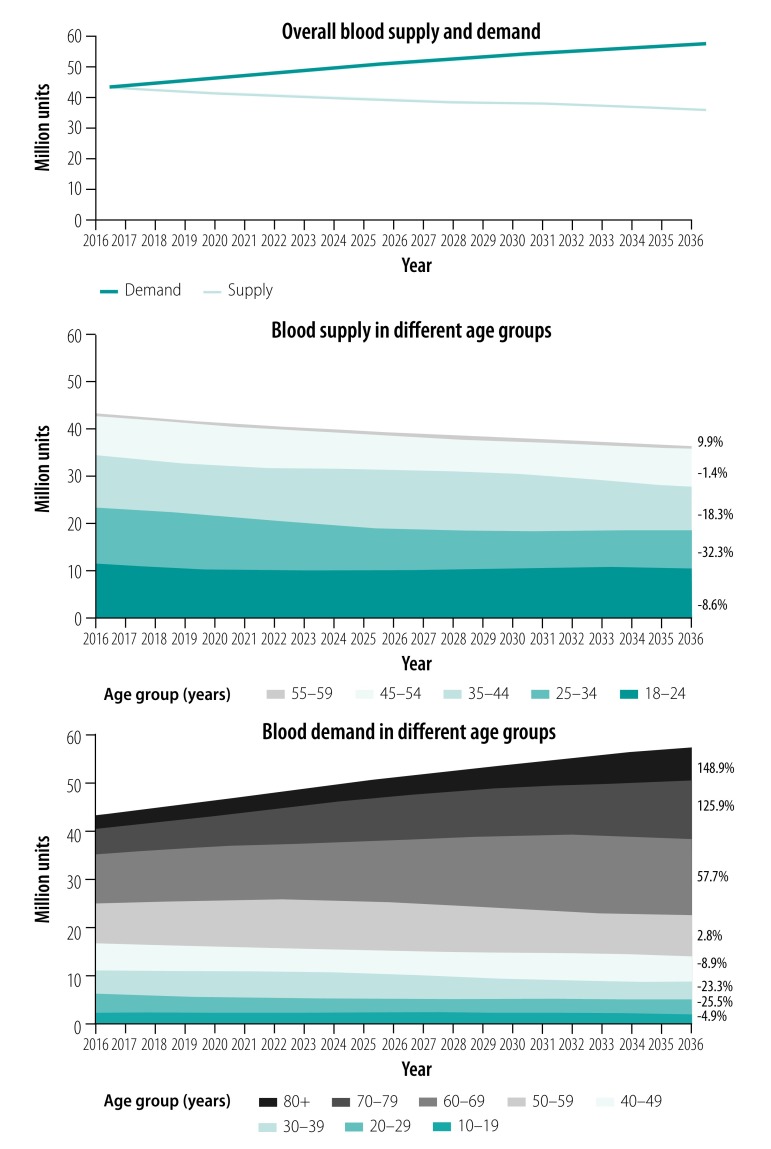
Predicted overall and age-specific blood supply and demand in China, 2016–2036

Notes: In the two panels showing data for different age groups, the percentages are the difference between 2036 and 2016 (baseline year). Percentage change in demand is not shown for the age group 0–9 years (−21.8%). One unit equals 200 mL blood.

The overall blood supply and demand predicted in the next 20 years, based on 2016 data (43.2 million units), are shown in Fig. 3. We estimated that blood supply would decrease after 2016, more than 5% by 2021 (to 40.9 million units) and reaching 10% by 2027 (to 38.9 million units). In contrast, the overall demand for blood increased sharply against the 2016 baseline, with an increase of about 10% by 2021 (to 47.2 million units) and 20% by 2027 (to 51.8 million units). By 2036, we estimated the blood supply would be 36.3 million units (a decline of 16.0% from 2016) and blood demand would be 57.5 million units (an increase of 33.1%), indicating a potential shortage of 21.2 million units (36.9% of the demand).

The most substantial decrease in supply (32.3%) will occur in the age group 25–34 years, from about 11.8 million units in 2016 to 8.0 million units in 2036. This decrease roughly corresponds to the sharp decrease in the size of this age group, from those born in the 1980s (236 million) to those born in the 1990s (172 million) and 2000s (160 million). Although the blood supply in the age group 55–59 years is estimated to grow by 9.9% (43 962/445 073) from 2016 to 2036 because of the 1960s birth boom, its effect on the overall supply trend is very small because the absolute increase is small (from 445 073 to 489 036 units; Fig. 3).

There are two divergent trends of age-specific blood demand (Fig. 3). Blood demand in all age groups younger than 50 years is expected to fall during the projected years, ranging from -4.9% (92 492/1 899 179) in the 10–19-year age group to -25.5% (1 012 390/3 972 959) in the 20–29-year age group. However, demand in all groups aged 50 years or older will increase in the next 20 years, especially in those aged 70–79 years (from 5 379 852 to 12 150 150 units, a 125.8% increase) and 80 years or more (from 2 836 230 to 7 059 161 units, a 148.9% increase).

**Fig. 4:**
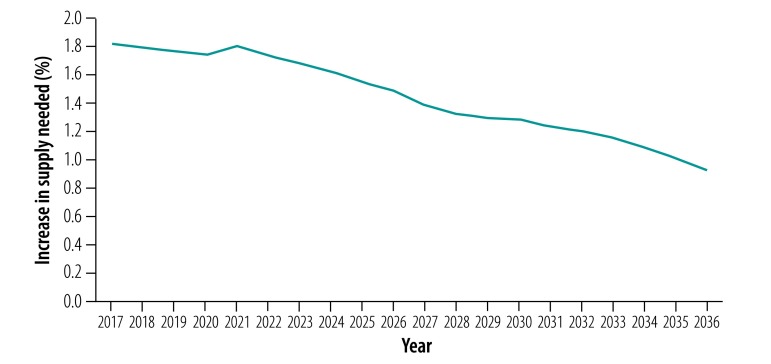
Predicted annual increase in blood supply needed to maintain a balance with demand in China, 2017–2036

Fig. 4 shows the increase in the overall blood supply needed to maintain a balance with demand during 2017–2036. The required increase in supply declined steadily over time, from 1.8% in 2017 to 1.5% in 2026 and to 0.9% in 2036, which is about half the increase in rate needed at the beginning of the period.

### Regional variations

**Fig. 5:**
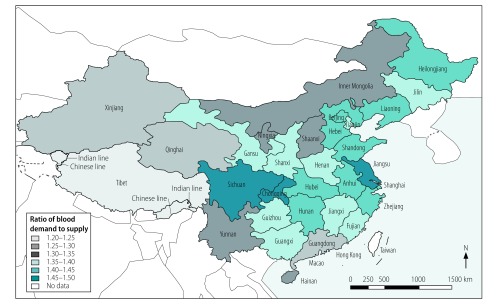
Predicted ratios of blood demand to supply in different regions of China in 2036

We predicted large variation between regions in the level of blood shortages projected for 2036 (Fig. 5). Regions with the highest demand/supply ratios (≥ 1.45) were Chongqing (a municipality in the south-west of the country), Sichuan (neighbouring Chongqing, whose population became older before achieving economic prosperity) and Jiangsu (the most affluent eastern province); these regions have the biggest proportions of older people (≥ 65 years). Regions with the lowest demand/supply ratios (≤ 1.30) were Guangdong (on the south-east coast, the most populous and one of the richest areas in China), Xinjiang (in the north-west), Qinghai (next to Xinjiang and one of the least developed regions) and Tibet (west of Qinghai).

### Sensitivity analysis

**Fig. 6:**
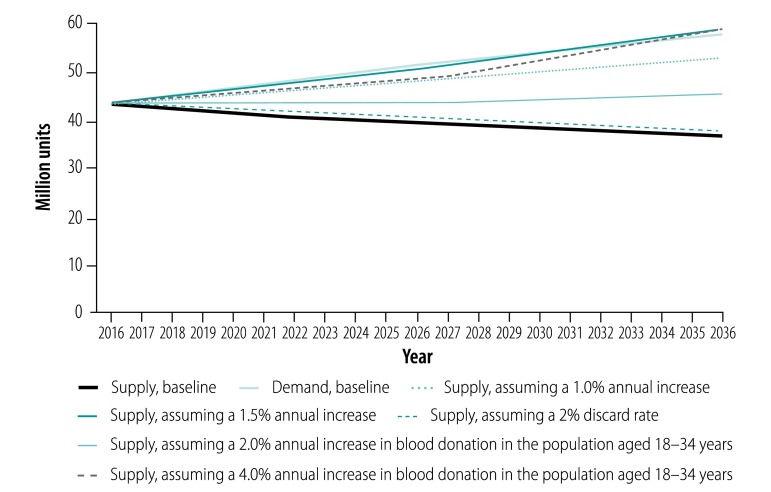
Sensitivity analysis of different assumptions of blood supply and demand in China, 2016–2036

Note: One unit equals 200 mL blood.

When we reduced the blood discard rate from 5% to 2% the overall supply–demand balance did not change greatly; however, we estimated that an additional 24.8 million units of blood would be available for clinical use over the next 20 years (Fig. 6).

A 2.0% annual increase in per capita blood donation in people aged 18–34 years would still be inadequate and would result in a shortage of 12.1 million units by 2036. An annual increase of 4.0% in this group alone would still be insufficient to reach a balance in supply and demand until 2035 and later (Fig. 6).

Increasing the blood supply, regardless of age group, seems to be the most effective approach. Maintaining an annual increase in blood supply of 1.0% would greatly reduce (though not offset) the imbalance with demand (a shortage of 4.8 million units by 2036). A 1.5% annual increase in supply would achieve a balance with demand by 2033, with a supply shortage of no more than 1.0 million units before this time. Between 2033 and 2036, supply would exceed demand by no more than 0.7 million units (Fig. 6).

## Discussion

China has the highest volume of annual blood collection and number of volunteer donors in the world.^25^ Nonetheless, because of demographic changes, we predict a decline in the blood supply and an increase in demand by 2036, relative to 2016. To maintain a nationwide balance in supply and demand for blood, an annual growth in supply is required (Fig. 4). Moreover, regional variation is estimated to grow and regions with large proportions of older people, will have an even greater demand than supply in 2036. These findings suggest a growing imbalance between blood supply and demand between regions, which requires immediate, strategic and ongoing action.

Our study results are based on population data from the World Population Prospects,^10^ and blood service data of the whole country.^13^ The use of world population data facilitates comparison of our results with those of other countries. For example, a shortage in blood supply of 25–40% in about 2035 has also been predicted in Canada,^5^ Iceland,^26^ Japan,^6^ and Switzerland,^27^ despite differences in modelling parameters and approach. To a certain extent, the regional variation shown in our study is representative of the situation worldwide. Therefore, sharing information and experiences between countries will help deal with problems with blood supply arising from ageing populations worldwide.

The study has limitations. The assumption of constant age-specific blood donation and transfusion frequencies over time is common in many demographic models^3^^–^^5^ but may not reflect the complex reality.^28^ For instance, overall blood donation did not decrease, but continued to rise during 2016–2018, as a response to the substantial efforts made in China to encourage blood donation.^29^ The value of this assumption is to inform investment in such efforts by estimating the potential influence of demographic shifts (a key predicable factor); we did not intend to model every possible scenario that is vulnerable to change and less quantifiable. For the same reason, we did not analyse potential changes in donation policy (e.g. expanding the eligible donor age^30^^,^^31^ and donation frequency,^32^ as adopted or considered in high-income countries but not yet in most regions of China). Currently, the donation policy in most places in China allows a donation of 200, 300 or 400 mL of blood at one time, and an interval between donations of not less than 6 months for whole-blood donations and not less than 28 days for platelet donation.

Blood demand may have been underestimated because we assumed it to be equal to blood supply in 2016 and is expected to be affected by future medical advances.^28^^,^^33^

Data on time trends in the blood supply,^27^^,^^34^ transition probabilities between donation frequencies in succeeding years,^6^ and retention rates of donors^34^ can be used to construct more sophisticated (and perhaps more precise) prediction models; however, such data were unavailable in our study. The effects of specialty hospitals (6642 hospitals in 2016),^35^ minority ethnic groups (120 million people, with low reported donation rates because of cultural beliefs),^36^ and sex differences in blood donation and consumption (e.g. more women in their 30s and 40s having a second child and higher risk of maternal haemorrhage with more births as a result of the new birth policy)^11^ were also not analysed in this study.

### Recommendations

Blood shortage is a problem requiring multidimensional solutions and close collaboration between researchers, blood bank staff, policy-makers and all of society. We can learn from efforts in high-income countries,^37^ and new solutions to sustain the blood supply continue to emerge.^38^^,^^39^ Instead of a discussion of specific solutions, we suggest the following overall strategies, which we consider of great importance to the future of blood service management in China.

#### Strategy I. Education

A voluntary, unpaid donation-based blood system in China is still in its early stages.^40^ Therefore, professional, public and early education should be strengthened across the country to encourage blood donation. In contrast to the more than 30 years’ experience in education on transfusion medicine in the USA,^41^ transfusion medicine only became a separate specialty in China in 2016.^13^ Only seven of over 2500 universities nationwide now offer undergraduate education in transfusion medicine. Accelerating professional education and developing qualified blood service teams are important, especially to train personnel on assuring the quality, safety and appropriate use of blood.^42^ In addition, increasing public awareness and helping more eligible adults to understand the importance of blood donation^43^^,^^44^ could greatly increase the number of blood donors and hence the blood supply. For example, the current blood donation rate among young people is much lower in China (30 donors per 1000 population) than in high-income countries (e.g. 116 per 1000 in Poland).^25^ With long-term problems in blood donation and an ageing population similar to China,^6^^,^^15^ Japan has set several good examples, especially early education of schoolchildren,^6^ the main blood donors of the future.

#### Strategy II. Tailored methods

The large regional variation in blood supply and demand in China is a unique challenge; thus, strategies to tackle the issue should vary accordingly. For example, given the very high predicted blood demand/supply ratios in Sichuan because of an ageing population,^45^ the age limit of healthy voluntary donors was increased from 59 to 65 years in 2019. Ensuring that only expired or unusable blood is discarded would also improve blood supply in Sichuan. In 2016, more than 10% of blood collected in the province was discarded,^13^ which could be compared with a discard rate of 1% in Jiangsu. In other regions of western China, despite less pressure from ageing populations,^11^ there is a rapid increase of blood demand, which calls for preparedness of blood services in equipment, facilities, staff, organization^40^ as well as technical support from the eastern regions of China as needed. Sentinel hospitals (as in the Republic of Korea)^46^ should be established in sparsely populated areas to better understand and cater to the needs of residents for blood services.

#### Strategy III. Multilevel coordination

China has considerable experience in network and systems construction, which could be used to improve coordination of blood services. First, cross-sectoral coordination: given the large blood shortage according to our projection, mechanisms should be established to strengthen transparency and communication between blood banks, hospitals and communities to match patient needs.^37^ Second, cross-regional coordination: even though 98.6% (8709/8831 units) of blood products were reallocated within provinces in 2016,^13^ increasing the movement of blood products across provincial borders may offset urgent shortages,^5^ especially in regions predicted to not be self-sufficient in future. Third, urban–rural coordination: population ageing in rural China is twice that in urban areas.^11^ As can be inferred from our sensitivity analyses on people aged 18–34 years, these young adults relocating to work in cities would create greater difficulties for blood services in rural areas and for their parents and the children left behind.^47^ Fourth, short-term coordination: although average predicted blood shortages are not high in provinces with high levels of imported labour (e.g. Guangdong), reliance on university students and migrant workers results in seasonal blood shortages during summer holidays and the annual celebration of the Chinese New Year,^40^ which is a special challenge requiring more flexible and coordinated solutions.
