# Corrigendum

**DOI:** 10.2471/BLT.20.100320

**Published:** 2020-03-01

In: Xiaochu Yu, Zixing Wang, Yubing Shen, Zhong Liu, Hongjie Wang, Shumei Zhang, et al. Population-based projections of blood supply and demand, China, 2017-2036. Bull World Health Organ. 2020 Jan 1;98(1):10–18

On page 14, Figure 5, should read as follows:

**Fig. 5:**
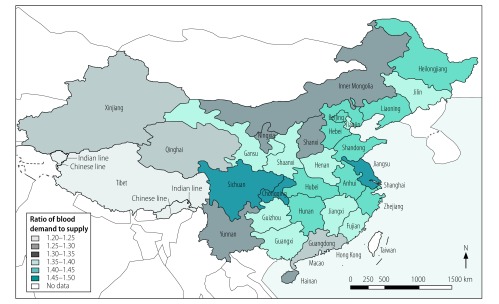
Predicted ratios of blood demand to supply in different regions of China in 2036

